# Process study of ceramic membrane-coupled mixed-cell fermentation for the production of adenine

**DOI:** 10.3389/fbioe.2022.969668

**Published:** 2022-08-10

**Authors:** Pengjie Sun, Changgeng Li, Yu Gong, Jinduo Wang, Qingyang Xu

**Affiliations:** ^1^ National and Local United Engineering Lab of Metabolic Control Fermentation Technology, Tianjin University of Science and Technology, Tianjin, China; ^2^ College of Biotechnology, Tianjin University of Science and Technology, Tianjin, China

**Keywords:** adenine, adenosine, ceramic membrane, *Bacillus subtilis*, nucleoside hydrolase

## Abstract

In order to solve the problems of high complexity, many by-products, high pollution and difficult extraction of the existing adenine production process, in this study, ceramic membrane-coupled mixed cell fermentation was used to produce adenine while reducing the synthesis of by-products and simplifying the production process of adenine. Nucleoside hydrolase (encoded by the *rihC* gene) was used to produce adenine by coordinated fermentation with the adenosine-producing bacterium *Bacillus Subtilis* XGL. The adenosine hydrolase (AdHy)-expressing strain *Escherichia coli* BL21-AdHy was successfully employed and the highest activity of the crude enzyme solution was found by orthogonal experiments at 170 W power, 42% duty cycle, and 8 min of sonication. The highest AdHy activity was found after 18 h of induction incubation. *E. coli* BL21-AdHy was induced for 18 h and sonicated under the above ultrasonic conditions and the resulting crude enzyme solution was used for co-fermentation of the strain and enzyme. Moreover, 15% (v/v) of the AdHy crude enzyme solution was added to fermentation of *B. subtilis* XGL after 35 h. Finally, the whole fermentation system was dialyzed using coupled ceramic membranes for 45 and 75 h, followed by the addition of fresh medium. In contrast, the AdHy crude enzyme solution was added after 35, 65, and 90 h of *B. subtilis* fermentation, with three additions of 15, 15, and 10% of the *B. subtilis* XGL fermentation system. The process was validated in a 5 L fermenter and 14 ± 0.25 g/L of adenine was obtained, with no accumulation of adenosine and d-ribose as by-products. The enzymatic activity of the AdHy crude solution treated with ultrasound was greatly improved. It also reduced the cellular activity of *E. coli* BL21-AdHy and reduced effects on bacterial co-fermentation. Membrane-coupled dialysis solved the problem of decreased yield due to poor bacterial survival and decreased viability, and eliminated inhibition of the product synthesis pathway by adenosine. The batch addition of crude enzyme broth allowed the continuous conversion of adenosine to adenine. This production method provides the highest yield of biologically produced adenine reported to date, reduces the cost of adenine production, and has positive implications for the industrial production of adenine by fermentation. And it provides a reference for producing other high-value-added products made by fermentation.

## Introduction

Adenine is one of the four basic bases of nucleic acids, which are essential molecules for life and evolution (Zhao et al., 2018). It is used in the treatment of granulocyte deficiency and neutropenia (Takeuchi et al., 2015) and can indirectly provide energy to red blood cells during blood storage (Paglia et al., 2016; Hess and Greenwalt, 2005). Adenine is also an intermediate in the production of many drugs and is used in the synthesis of phytohormone I, vitamins, adefovir, and vinegar. Owing to the wide range of applications of adenine, its use has been increasing worldwide in recent years.

Nowadays, the main methods for producing adenine are chemical synthesis and enzymatic catalysis. Li et al. (2016) prepared adenine from malononitrile and thiourea by cyclization in the presence of sodium alcohol, followed by oxidation, nitrosation and reduction; You et al. (2013) prepared adenine from acetyl hypoxanthine by chlorination and amination. Both of the above methods for preparing adenine by chemical synthesis suffer from harsh reaction conditions, complex reaction steps and environmental pollution. Lv and Fu (2008) prepared adenine by hydrolysis of adenosine at high temperature and pressure at 170–210°C. Yue et al. (2020) used adenosine as raw material and hydrolyzed adenosine into adenine and d-ribose under the combined action of adenosine hydrolase and neutral protease. Xie et al. (2018) successfully constructed an adenine-producing strain by introducing the gene encoding adenosine hydrolase, *rihC*, from *E. coli* into the original adenosine-producing bacteria. However, the fermentation cycle was long, with 44 h of fermentation production and a yield of only 6.38 g/L.

The rapid progress in the field of biotechnology constantly attracts new methods and solutions for further development of bioprocess performances. In the last decade, ultrasound has been widely used for enzyme-catalyzed biotransformations to enhance the reaction process and obtain higher product yields in a short period, as ultrasound can enhance enzyme activity by altering favourable conformational changes of proteins without changing their structural integrity. However, the tolerance of enzymes to ultrasound depends on the physiological properties of the enzymes themselves. Ladole et al. (2017) showed a 3.6-fold increase in the catalytic activity of cellulase compared to the control by incubating the enzyme at 24 kHz, 36 6 W power and 6 min ultrasound. *Pseudostelium antarcticum* lipase B activity increased 1.5-fold at 22 kHz, 15.48 W cm ^−2^ and 66.67% duty cycle (Nadar and Rathod, 2017). In recent years, the coupling of fermentation systems with other biotechnologies has been used as a new form of fermentation to increase product yields. He et al. (2021) increased the activities of three key enzymes in glycolytic metabolism (hexokinase, phosphofructokinase and pyruvate kinase) by adding an ultrasound device to the fermentation of brewer’s yeast for ethanol production at 280 w/L. 59.02, 109.05 and 87.27%, respectively, and the ethanol production increased by 30.79%. Coupling a butterfly centrifuge during the l-tyrosine fermentation process and cycling the fermentation system to fractionate the fermentation process increased l-tyrosine yield by 44.1% (Li et al., 2021).

In this study, a bacterial and enzyme-linked ceramic membrane co-fermentation method was used for the preparation of adenine. The conversion of adenosine to adenine and d-ribose was first achieved by adding AdHy crude enzyme solution to the production of adenosine by fermentation in *B. subtilis*. The batch addition of AdHy crude enzyme solution ensured the continuous conversion of adenosine. The d -ribose is involved in cellular energy metabolism (Teitelbaum et al., 2006) and can also be reused as a high-quality carbon source for *B. subtilis XGL*, thus reducing sugar consumption. Continuous production of adenosine is ensured by coupling ceramic membranes and adding nutrient flow to enhance strain viability and release the product from inhibition of the synthetic pathway. Nowadays, with the rise of biological methods, most nucleosides can be produced by biological processes. However, direct fermentation to produce adenosine has not been reported due to the strict feedback regulation of the purine synthesis pathway by adenosine. This method combines enzymatic and fermentation methods to successfully produce adenine by reusing d-ribose’s by-product. Compared with the previous adenine production method, the present method has the advantages of high adenine yield, low environmental pollution, low by-products and low production costs, and it is suitable for the industrial production of adenine.

## Materials and methods

### Strains, plasmids, primers, and media

In this study, *E. coli* DH5α was used as a host strain for cloning and plasmid construction. *E. coli* BL21 was used as a host strain for enzyme expression, and pET-28a plasmid was used as an expression vector bearing the selectable kanamycin sulfate screening marker. Recombinant plasmids were constructed using the One Step Cloning Kit from Vazyme Biotech Co., Ltd (Nanjing, China). *E. coli* cells were cultured and screened on LB medium (1% peptone, 0.5% yeast powder, 1% NaCl) at 37 °C with the addition of 100 μg/ml ampicillin. The *E. coli* BL21-AdHy strain induction expression medium is presented in Supplementary Table S1. In this study, *B. subtilis* XGL (this isolate was screened by UV mutagenesis and was histidine- and xanthine-deficient) was used as the adenosine producing strain in *B. subtilis* XGL medium (see Supplementary Table S2 for the specific formulation), and the medium added after dialysis was the same (all reagents purchased from Sinopharm Chemical Reagent, Tianjin, China). The strains and plasmids used in this study are listed in Supplementary Table S3 pET-28a identification primers synthesized by Genewiz (Jiangsu, China) are listed in Supplementary Table S4. Plasmid extraction was performed using the plasmid rapid extraction kit from Omega Bio-tek Co. (Shanghai, China).

### Construction of *E. coli* BL21-AdHy

We used pET-28a with a T7/lac-inducible strong promoter (induced by Isopropyl *β*-D-1-Thiogalactopyranoside (IPTG); for initiation). The nucleoside hydrolase used in this study (encoded by the *rihC* gene, GenBank ID: WP112886990.1), designed with the *rihC* fragment in pET-28a that has overlapping parts with the enzymatic sites EcoRⅠ and HindⅢ, was synthesized by Genewiz (Jiangsu, China). Details of plasmid and strain construction are given in Arivett et al. (2014).

### Culture of *E. coli* BL21-AdHy

*E. coli* BL21-AdHy cells were transferred from glycerol preservative tubes into strain activation medium (see Supplementary Table S1) for activated culture (two generations of activation) with the temperature maintained at 37 °C. Activated strains were inoculated by aseptic manipulation into 5 L fermenters and incubated at a pH of approximately 6.7–7.0, a temperature of 37 °C with 35–50% dissolved oxygen. The induction of AdHy expression was initiated by adding IPTG (0.1 mmol/L) within 4–5 h of incubation. (*E. coli* BL21-AdHy induction medium is presented in Supplementary Table S1).

Preparation of AdHy crude enzyme solution employed an ultrasonic bar with a power of 170 W and a frequency of 25 kHz was chosen to treat the bacteria, specifically, working its way through the process by working for 8 min with a duty cycle of 42%, during which time the temperature was controlled at 4°C and the final solution was stored in a 4°C tank for backup until the end of the overall fermentation process.

### Culture of *B. subtilis* XGL

*B. subtilis* XGL was inoculated from glycerol-preserved tubes into activation medium (two generations of activation culture) and incubated at 34°C for 10–12 h. The activated strains were transferred to seed jars at 34°C, pH 6.7–7.0, with 30–50% dissolved oxygen. When the cells reached the middle of the exponential growth phase (OD_600_: optical density at 600 nm; OD_600_ = 15–20), the seed culture was introduced into the fermenter as a 20% inoculum. Temperature was maintained at 34°C, with dissolved oxygen at 30–50% and pH 6.7–7.0 at the start of fermentation, with pH controlled at 6.4–6.7 from mid-fermentation (OD_600_ > 30). After 10 h of fermentation, flow-through of complex nutrients at a rate of 0.3 g/L/h was initiated (detailed composition of the medium is listed in Supplementary Table S2).

### Bacterial and enzyme mixed coupling ceramic membrane fermentation

**FIGURE 1:**
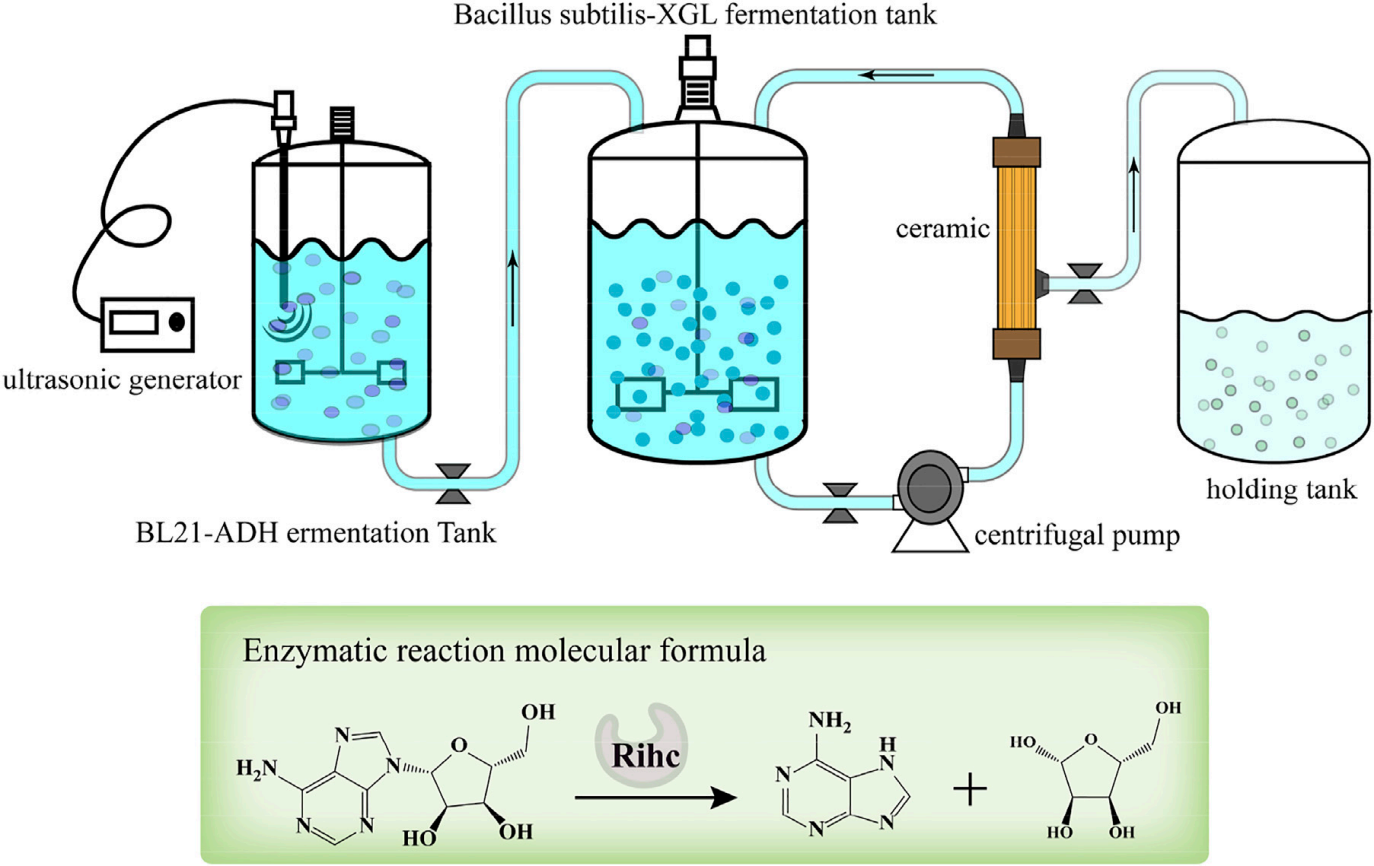
Schematic diagram of the production of adenine by ceramic membrane coupled mixed cell fermentation.

The hybrid coupled ceramic membrane fermentation system for bacteria and enzymes consisted of a fermenter (Shanghai Baoxing Biological Equipment Engineering Co., Ltd., Shanghai, China), a multi-frequency power ultrasound device (Hebei Handan He Tao Machinery Technology Co., Ltd., Hebei Handan, china) and a ceramic membrane circulation system. The ceramic membrane circulation system consisted of a centrifugal pump (Nanfang Pump Co., Ltd., Jiangsu, China), a ceramic membrane (Shanghai Gaubire Environmental Engineering Co., Ltd., Shanghai, China) and a storage tank (Shanghai Baoxing Biological Equipment Engineering Co., Ltd., Shanghai, China). Adding AdHy crude enzyme solution to *B. subtilis* fermentation system, and two dialysis operations were performed during fermentation. The pH was maintained at 6.4–6.6 (pH adjusted by flow-through addition of ammonia), with dissolved oxygen at 40–60% and a temperature of 36°C. The process of coordinated dialysis fermentation with biomass is illustrated in Figure 1.

### Analytical methods

#### Detection of fermentation process

Determination of biomass: the optical absorbance at 600 nm was measured using a UV spectrophotometer (Beijing Pu-analysis General Instrument Co., Ltd.,Beijing, China) after diluting the sample10-100 times.

Determination of residual sugar content during fermentation: 1 ml of fermentation broth was sampled, centrifuged at 13,000 r/min for 2 min, the supernatant was diluted 100 times and the sugar content of the fermentation broth was determined using an SBA-40E biosensor (Institute of Biology, Shandong Academy of Sciences, Jinan, China).

#### Verification method of strain construction

Colony PCR amplification validation method: The single colonies cultured overnight were inoculated into 15 μL colony PCR system for reaction. At the end of the reaction, agarose gel electrophoresis was used to verify the size of the band (refer to Petersen and Møller, 2001, for specific steps.).

SDS-PAGE analysis: refer to (García-Fraga et al., 2015) for specific procedures. Nucleic acid and protein electrophoresis apparatus (Beijing Liuyi Instrument Factory, China).

#### AdHy enzyme activity identification

Enzyme activity assay method: 2 ml of bacterial solution was sampled after ultrasonication and 100 μL was added to 10 ml of enzyme reaction system (adenosine 15 g/L, PBS buffer 4.9 ml) and the reaction was terminated by boiling for 20 min, after which the adenine yield was determined by high performance liquid chromatography (HPLC).

Definition of enzyme activity unit: The amount of enzyme required to catalyse the production of 1 mol/L of adenine from the substrate adenosine in 1 min under standard enzyme activity assay conditions was defined as one enzyme activity unit, i.e. 1 U.

### Product detection method

Product determination:Determination of products by HPLC(Shimadzu, Japan), chromatographic conditions were as follows; Liquid chromatography separation conditions: column temperature 30 °C, detection wavelength 259 nm, total mobile phase flow rate 0.8 ml/min, mobile phase 10% (v/v) acetonitrile and 0.05% (v/v) trifluoroacetic acid, column: Kromasil C18 column (250 mm × 416 mm×5 μm USA).

## Results and discussion

### Construction of *E. coli* BL21-AdHy

#### Pet-28a-*rihC* plasmid construction

**FIGURE 2:**
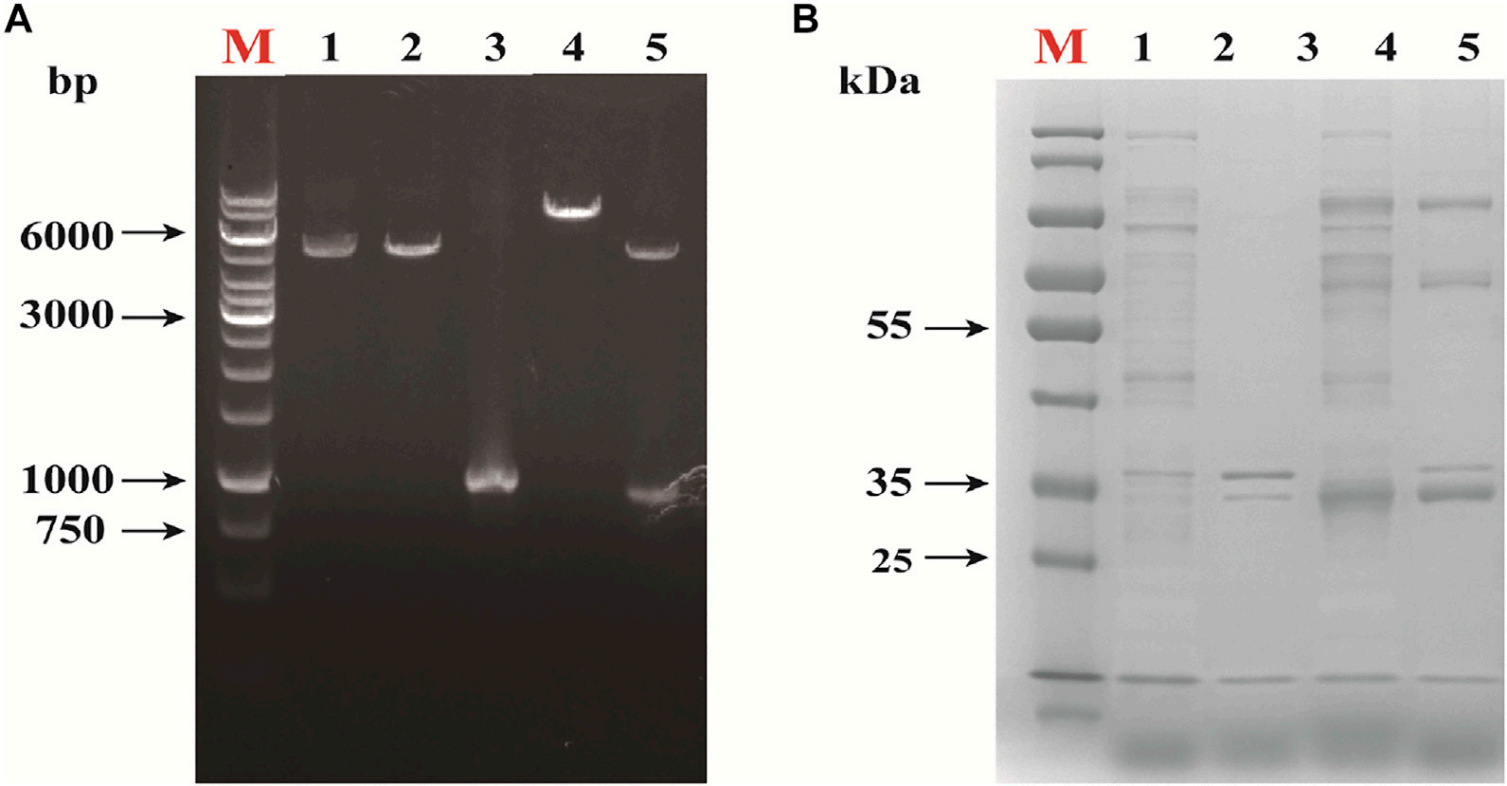
Alidation of Recombinant Expression of Nucleoside Hydrolase *rihC*. **(A)**Restriction map of recombinant plasmid. M: DNA marker; 1:*Eco*R Ⅰ single enzyme digestion of pET-28a; 2:pET-28a was digested by *Eco*R Ⅰ and *Hin*d Ⅲ. 3: target gene fragment; 4: *Eco*R I single enzyme digestion of pET-28a-*rihC*; 5: pET-28a-*rihC* was digested with *Eco*R I and *Hin*d Ⅲ. **(B)** SDS-PAGE analysis of the recombinant protein. M: protein marker; 1: *E. coli* pET-28a cell lysate supernatant sample; 2: *E. coli* pET-28a cell lysate precipitation sample; 3: *E. coli* pET-28a-*rihC* cell lysate supernatant sample; 4: Sedimentation in cell lysate of *E. coli* pET-28a-*rihC.*

The results of colony polymerase chain reaction (PCR) on the successfully constructed *E. coli* DH5α cells containing pET28a-*rihC* recombinant plasmid are shown in Figure 2A. The expected bands appeared around 1,221 bp; the recombinant plasmid was verified by enzymatic digestion using EcoRⅠ and HindⅢ, as shown in Figure 2A. The bands appeared around 5,350 bp and 912 bp. The linearized plasmid and the target gene showed consistent bands, which indicated that the Pet-28a-*rihC* plasmid was successfully constructed, with two verifications.

#### Construction of E. coli-BL21-AdHy

The *E. coli* BL21-AdHy strain was subjected to SDS-PAGE analysis and the results are shown in Figure 2B. The supernatant of the homogenized bacteria showed the expected band (around 33 kDa), demonstrating that the protein could be stably expressed by the recombinant strain.

### Effects of incubation time and ultrasound treatment on enzyme and cell activity

#### Effect of incubation time on enzyme expression

**FIGURE 3:**
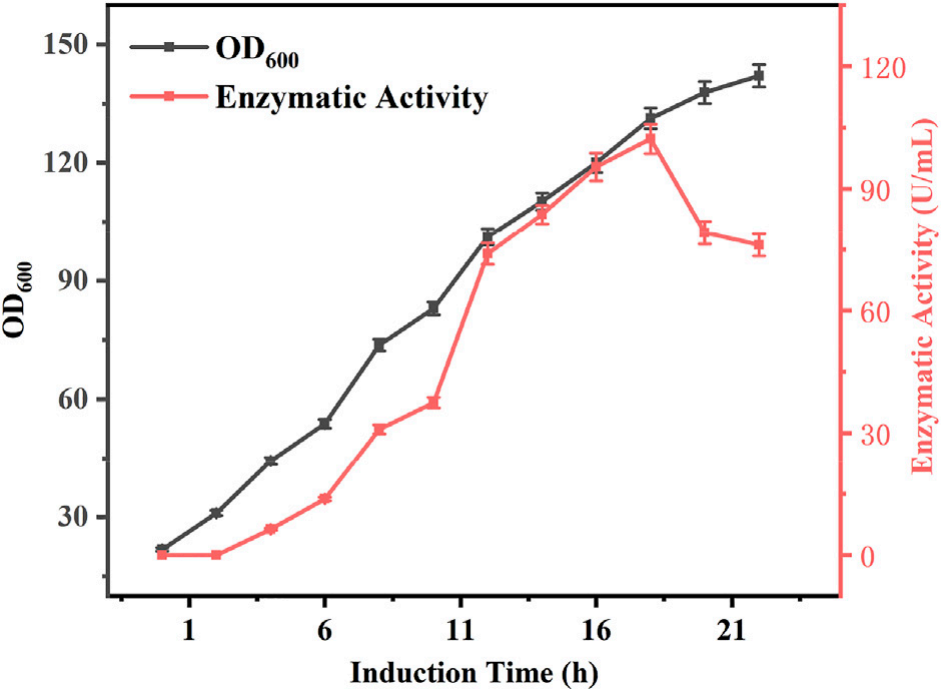
Changes in enzyme activity and biomass during induction culture.

During induction culture of the bacteria, the enzyme activity is positively correlated with the incubation time of the bacterium for a certain period of time. However, prolonged incubation periods can also affect enzyme activity. To investigate the relationship between induction culture time and enzyme activity, *E. coli* BL21-AdHy were induced in 5 L fermenters and their biomass and enzyme activity were examined, the results of which are shown in Figure 3. At the beginning of induction, the biomass was 21.8, and the enzyme activity was 0 U/mL. Both biomass and enzyme activity increased significantly with the extension of induction time, and the enzyme activity was the highest at 18 h. At this time, the biomass was 131.2, and the enzyme activity was 102.2 U/mL. With the passage of time, the biomass still increased, whereas enzyme activity exhibited a decreasing trend. Analysis suggested that the increase in enzyme activity during the early stages was due to the lack of induced expression of the enzyme and the low biomass per unit volume, such that enzyme activity continued to increase in the first 18 h. It is known that longer induction times increase protein expression, but may also jeopardise protein stability or induce protein hydrolysis (García-Fraga et al., 2015); alternatively, studies have shown that at high biomass levels, the availability of dissolved oxygen decreases and various substances affecting protein stability are produced (Olaofe et al., 2010; Hu et al., 2015), leading to a decrease in enzyme activity. However, after 18 h of induction culture, AdHy enzymatic activity tended to decrease with increasing induction time. Therefore, an 18 h period for induction culture of *E. coli* BL21-AdHy was selected to produce a crude enzyme broth.

#### Effects of different ultrasound treatment modes on enzyme activity

**FIGURE 4:**
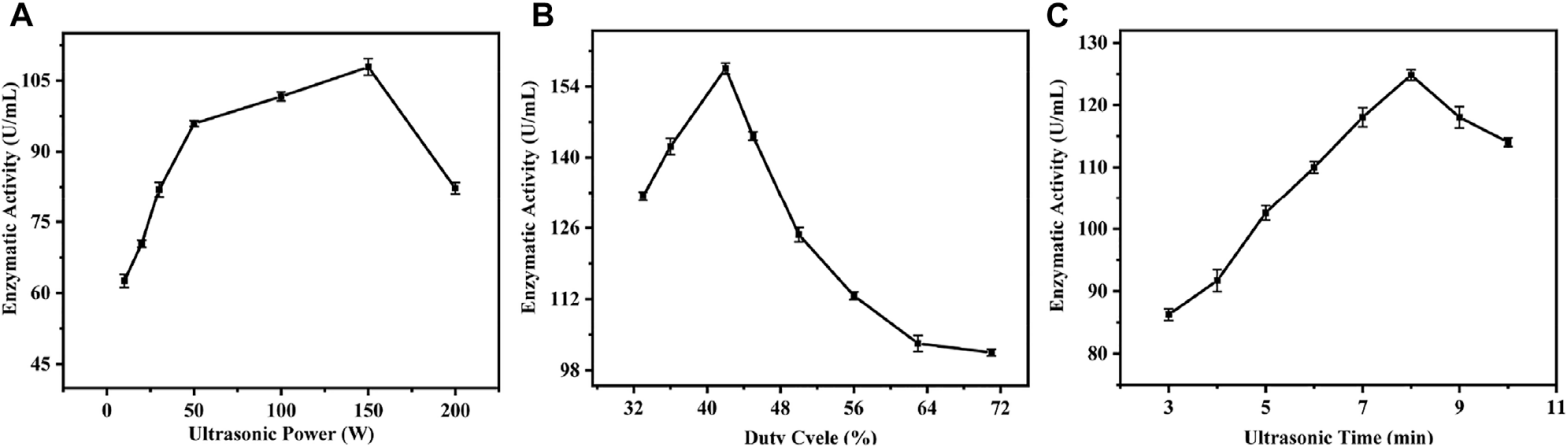
Single factor experiment of ultrasonic parameters. **(A)** Effect of ultrasonic power on enzyme activity; **(B)** Effect of Duty Ratio on Enzyme Activity; **(C)** Effect of ultrasonic time on enzyme activity.

Ultrasound is now widely used for cleaning, lithotripsy, and sterilization because of its good directionality and penetrating power. Extreme ultrasound treatment can cause loss of enzyme activity (O'Donnell et al., 2010), whereas low frequency ultrasound can enhance enzyme activity by altering favorable conformational changes in proteins without altering their structural integrity (Duan et al., 2011). The tolerance of enzymes to ultrasound depends on the physiological properties of the enzyme and ultrasound parameters, such as ultrasound power, duty cycle, and treatment time, which can directly affect catalytic activity of the enzyme (Kentish and Ashokkumar, 2011; Goncalves et al., 2015). Therefore, in order to investigate the effects of different sonication conditions on the activity of nucleoside hydrolase crude enzyme solution, sonication time, sonication power, and duty cycle were used here as conditions for investigation, and specific experiments and experimental results are shown in Figure 4.

To investigate the effects of different sonication power on the activity of crude enzyme solution, a duty cycle of 62% and a sonication time of 5 min were chosen, and different sonication powers were selected for single-factor experiments. The experimental results are shown in Figure 4A. Under low power sonication, the activity of crude enzyme solution increased with increasing sonication power; the highest activity of 107 U/mL was achieved under the treatment condition of 150 W. However, with further increases in power, the activity showed a trend of weakening. Analysis showed that low-power ultrasound could induce stable cavitation in the surrounding liquid, and the generation and extinction of cavitation bubbles can cause oscillations in the liquid (Rao and Rathod, 2014; Gonçalves et al., 2015), which in turn causes changes in the structure of the cell membrane, increasing its permeability to some extent and even disrupting it, causing enzyme molecules to flow from inside the cell to outside the cell (Zhang et al., 2021), and ultrasound produces benign changes in the structure of enzyme molecules (Wang et al., 2012), leading to further increases in enzyme activity. As a result, enzyme activity tends to increase. However, with further increase in ultrasound power, the enzyme activity shows a gradual decrease, which may be due to the high power of ultrasound, which also carries higher energy and displays significant shearing in the surrounding liquid (Neslihan et al., 2006), in which case peptide chains of the enzyme molecule are disrupted, leading to loss of enzyme activity (Huang et al., 2017).

To investigate the effects of different duty cycles on the activity of the crude enzyme solution, ultrasound power of 100 W and ultrasound time of 5 min were selected, and eight different gradients in the duty cycle of 38–83% were selected for single-factor experiments, and the results are shown in Supplementary Figure S4B. As the duty cycle increased, the activity of the crude enzyme solution gradually increased and reached the highest level of 157 U/mL at 42% duty cycle; with further increase in the duty cycle, the activity showed a decreasing trend. This increase in activity is presumed to be due to the increase in exposure time of the enzyme molecules undergoing sonication and the effective modification of the enzyme (Nadar and Rathod, 2017) and was highest at 42% duty cycle; however, further increases in duty cycle revealed decreasing enzyme activity. This is presumed to be due to the fact that higher duty cycles result in greater heat being generated around the enzyme molecules, and the higher heat generation causes a loss of enzyme activity (O'Donnell et al., 2010).

In order to investigate the effects of different sonication times on the activity of the crude enzyme solution, the sonication power was chosen to be 100 W, the duty cycle was 63% and eight different treatment times were selected between 3 and 10 min for single-factor tests. The results are shown in Supplementary Figure S4C. The activity of the crude enzyme solution gradually increased with increasing sonication time, reaching a maximum of 124 U/mL after 8 min of treatment. It is hypothesized that this trend is caused by the duration of exposure of the enzyme molecule to sonication, and the effective modification of the enzyme molecule by such treatment (Nadar and Rathod, 2017).

#### Optimization of orthogonal experiments

In order to obtain higher enzymatic activity, the orthogonal experiments of L_9_ (3^3^) were performed to further optimize the sonication parameters by combining the previous single-factor experiments of sonication power, sonication time and duty cycle. The specific experiments and results are shown in Supplementary Table S5. The experimental data showed that the enzyme activity of the crude enzyme solution was the highest at 207 U/mL when the power was 170 W, the duty cycle was 42%, and the treatment time was 8 min.

#### Changes to *E. coli* BL21-AdHy under ultrasound treatment

**FIGURE 5:**
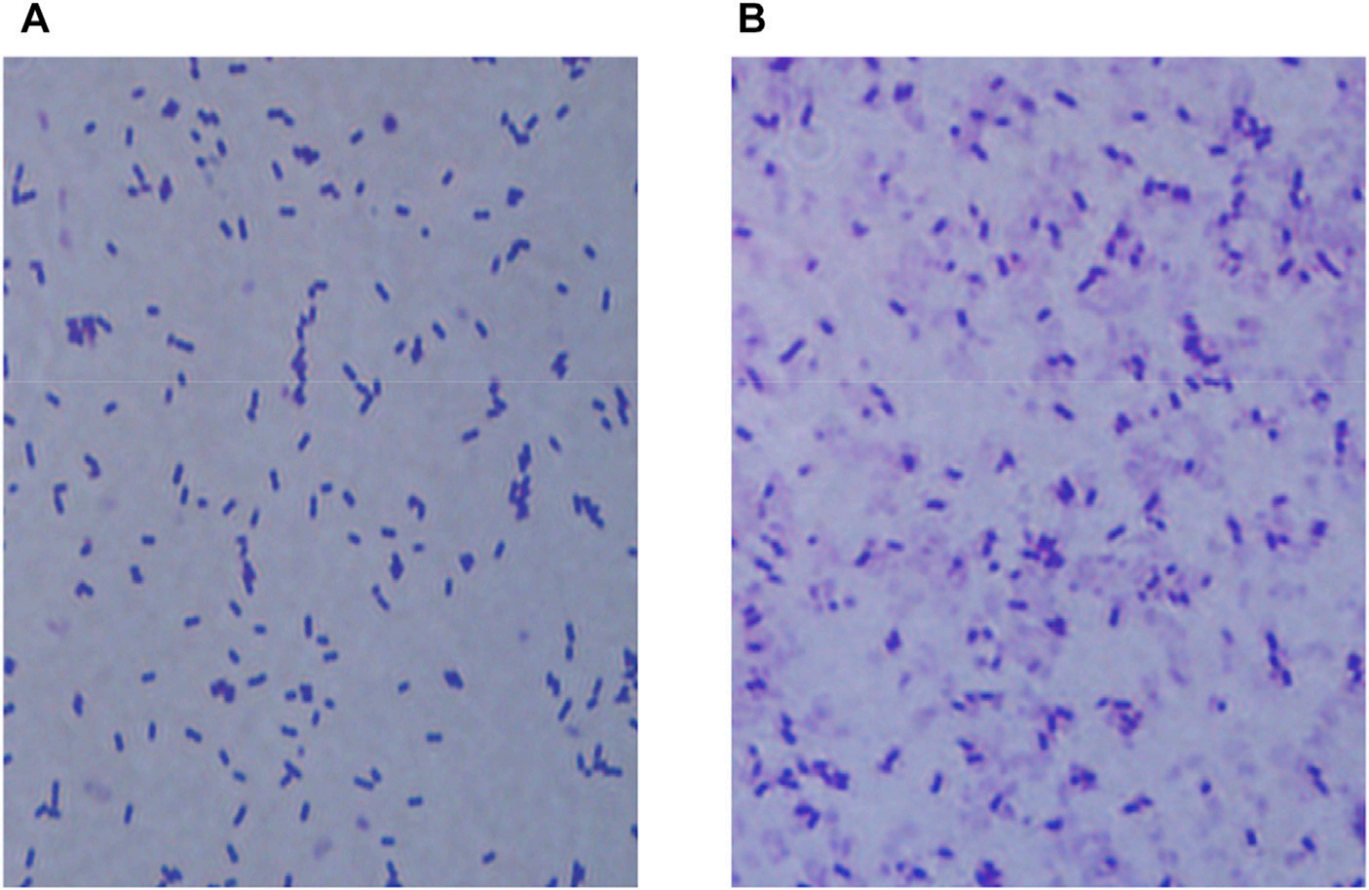
Morphological changes in E. coli BL21-AdHy under optimized ultrasound conditions. **(A)** Normal bacterial morphology; **(B)** Changes in bacterial morphology under ultrasound treatment.

Normal and sonicated bacteria were stained separately using Gram staining and observed by electron microscopy (100 ×) for comparison (as shown in Figure 5). It was hypothesized from the cell morphology that ultrasound treatment caused great structural damage to the cell membrane of *E. coli* BL21-AdHy, which in turn led to a significant decrease in cellular activity. The detrimental effect on the synergistic fermentation of bacteria and enzymes due to the addition of crude enzyme solution was reduced.

### Synergistic fermentation of bacteria and enzymes coupling ceramic membranes to produce adenine

#### Effects of timing of crude enzyme solution addition on synergistic fermentation of bacteria and enzymes

**FIGURE 6:**
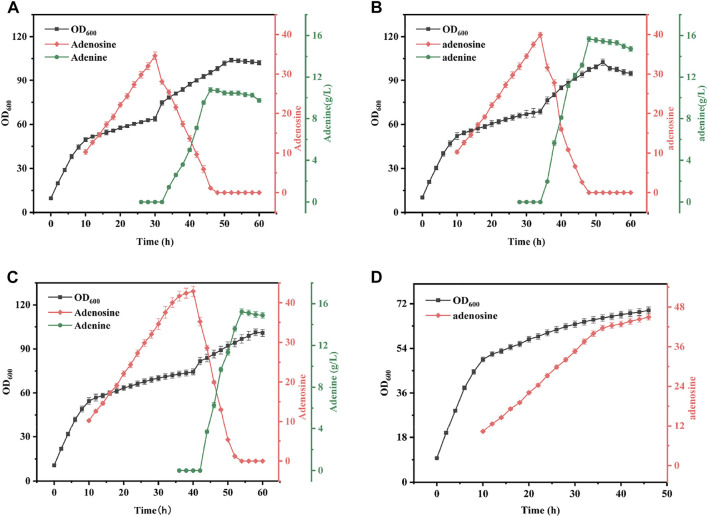
Effect of Different Mixing Time on Fermentation Results. Mixed ratio: 13% of *B. subtilis* XGL fermentation system. **(A)**: Changes of biomass, adenosine and adenine production during 30 h mixing operation; **(B)**: Changes of biomass, adenosine and adenine production during 35 h mixing operation; **(C)**: Changes of biomass, adenosine and adenine production during 40 h mixing operatio; **(D)**: Changes of biomass and adenosine production during normal fermentation.

In this study, it was found that mixing a certain percentage of sonicated AdHy crude enzyme solution during the fermentation production of *B. subtilis* XGL resulted in the enzymatic conversion of the original product adenosine to a new product: adenine. To investigate the effect of addition time on adenine yield, we added 13% (v/v) of AdHy crude enzyme solution at 30, 35, and 40 h of *B. subtilis* fermentation, respectively, and the results are shown in Figure 6. When the mixing time was 30, 35, and 40 h, the final adenine yields reached 9.73, 13.68, and 14.90 g/L, respectively. Note that the final yield was not the highest and adenosine did not accumulate. There was a high net increase in biomass after mixing, with a decreasing trend over time. Adenine production was highest when all adenosine present was transformed, but also declined slowly over time and production of *B. subtilis* XGL was found to have been terminated. Analysis showed that adenine production increased with increasing mixing time; this was due to the amount of adenosine substrate increasing with increasing fermentation time and therefore adenine production would increase with increased mixing time. The increase in biomass per unit time was further enhanced by the addition of sonicated crude enzyme solution. This is probably due to the fact that *B. subtilis* XGL was in the middle to late stages of growth when bacterial viability and growth rates are significantly reduced (Xu et al., 2021), and that the sonicated crude enzyme solution contains more *E. coli* fragments. Therefore, the crude enzyme solution contained nucleic acids and inorganic salts and sugars, which to some extent can act as high-quality complex nutrients, thus increasing the late growth rate of the bacteria. Eventually, the production of adenine declined slowly and the production of *B. subtilis* XGL stagnated. This is because adenine is involved in the synthesis of DNA and RNA and can therefore be used by bacteria but can only be consumed in small quantities due to the late stage of fermentation. The stalled production of *B. subtilis* XGL is due to feedback inhibition of the entire purine synthesis pathway by adenine (Smith et al., 1994; Rolfes, 2006) and when adenine reaches high concentrations, the purine synthesis pathway will also cease to function and therefore the bacterium will no longer produce adenosine. For reasons of bacterial growth, adenosine production efficiency and time involved, a 35-h mixed operation was chosen for this study.

#### Effects of crude enzyme solution addition ratio on synergistic fermentation of bacteria and enzymes

**FIGURE 7:**
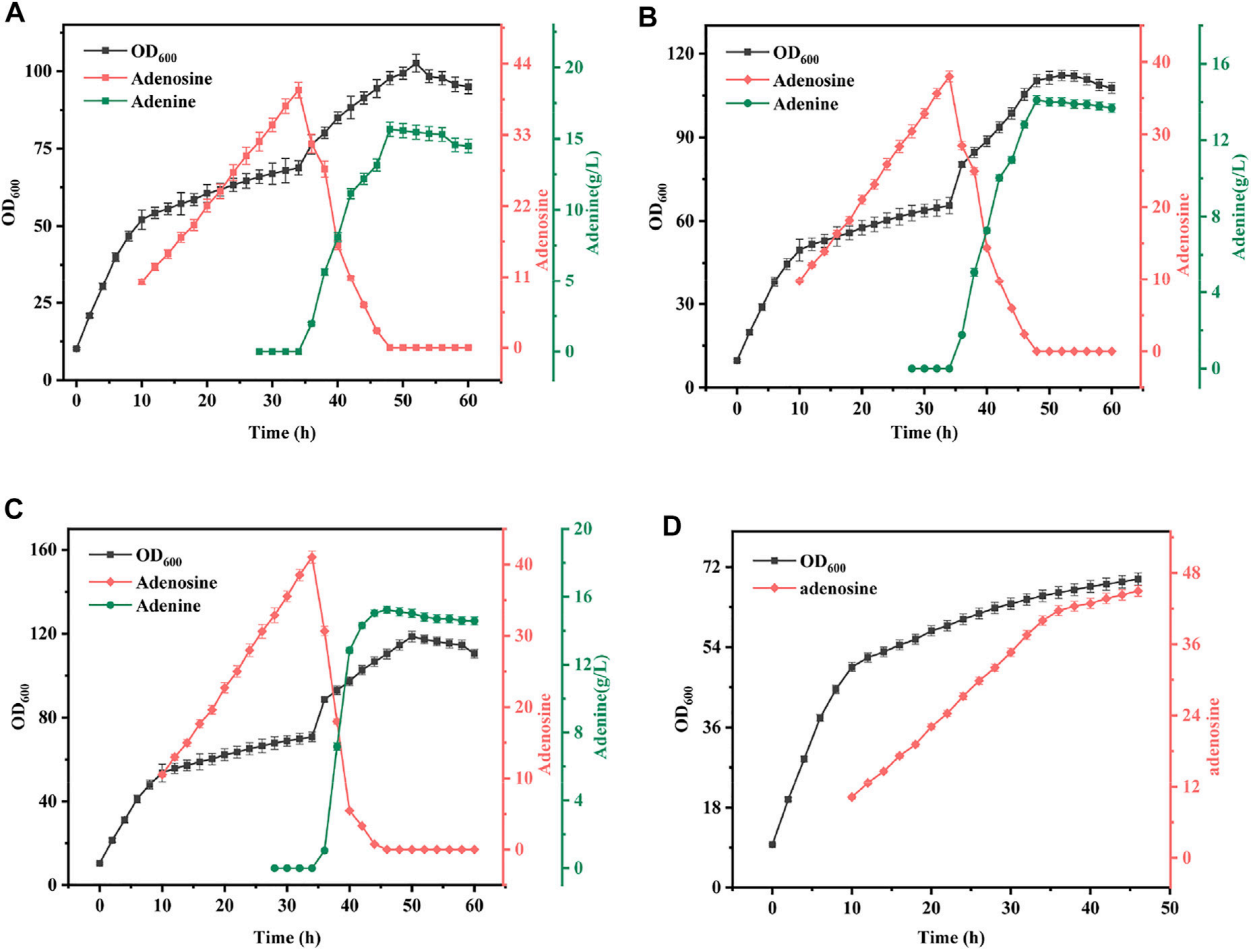
Effect of Different Mix Proportion on Fermentation Results. Mixed time: *B. subtilis* XGL fermented to 35 h **(A)**: When the mixed ratio was 6% of *B. subtilis* XGL fermentation system, the changes of biomass, adenosine and adenine production were observed; **(B)**: When the mixed ratio was 13% of *B. subtilis* XGL fermentation system, the changes of biomass, adenosine and adenine production were observed; **(C)**: When the mixed ratio was 20% of *B. subtilis* XGL fermentation system, the changes of biomass, adenosine and adenine production were observed; **(D)**: Changes of biomass and adenosine production during normal fermentation.

The mixing ratio is another key parameter in the synergistic fermentation of bacteria and enzymes and has a critical role in the conversion of adenosine. To investigate the effect of mixing time on the synergistic fermentation of bacteria and enzymes, in this study, crude enzyme solutions of 6, 13, and 20% were added to *B. subtilis* XGL fermentation after 35 h. The specific experimental results are shown in Figure 7. The conversion rate of adenosine to adenine was rapid as the mixing ratio increased. With the 6% addition, 12 h were required to complete the conversion of adenosine, but at the 20% mixing ratio, only 8 h were required to complete the conversion of adenosine. The conversion efficiency also increased steadily, from 41% at 6–48% at a 13% mixing ratio. However, when the mixing ratio was further increased, the elevation in terms of conversion efficiency was limited. Analysis showed that with a certain substrate, the amount of enzyme added determined the rate of product production and therefore, When the addition amount increased, the conversion efficiency of adenine was improved. Moreover, as the conversion efficiency was greatly improved, adenine conversion could be completed in a short period of time and the consumption of adenine during the conversion process was reduced and the conversion rate was also augmented. Since there is a linear relationship between conversion efficiency, conversion rate, and mixing ratio, 15% was chosen as the best mixing ratio in subsequent experiments.

#### Bacterial and enzymatic co-fermentation with coupled ceramic membranes

**FIGURE 8:**
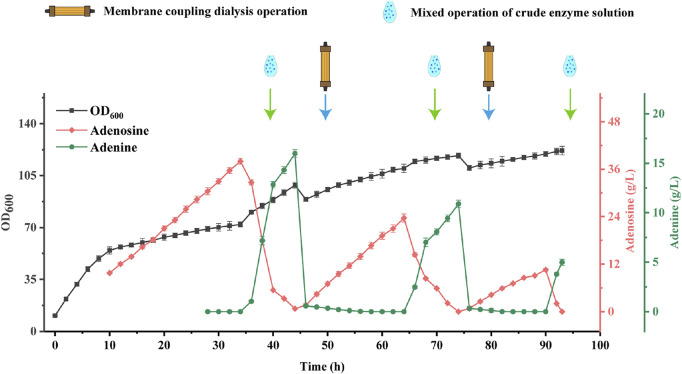
Changes of biomass, Adenosine and Adenine Production inBacterial and Enzyme Mixed Coupling Ceramic Membrane Fermentation.

In order to solve the problems of poor bacterial viability and low productivity in the late fermentation of *B. subtilis* XGL and stagnation of the overall purine metabolic pathway due to inhibition of adenine production in large quantities (Smith et al., 1994), we used a coupled ceramic membrane to dialyze the overall fermentation system with a flow-through of fresh nutrient solution, as shown in Figure 8.

In this experiment, the AdHy crude enzyme solution was added after 35, 65, and 90 h of *B. subtilis* fermentation, with three additions of 15, 15, and 10% of the *B. subtilis* XGL fermentation system. At 45 and 75 h, a coupled ceramic membrane dialysis operation was carried out, followed by refreshment with 60 and 40% of fresh medium. Adenosine production by *B. subtilis* XGL attained 38.72, 24.98, and 10.56 g/L after 35, 65, and 90 h. After addition of the crude enzyme solution, there was a stepwise increase in biomass. After coupled ceramic membrane dialysis, there was a stepwise decrease in biomass. The complete conversion of adenosine was completed around 10 h after the coupled ceramic membrane dialysis. This is presumably since membrane-coupled dialysis removes substances that are detrimental to the growth of bacteria in the later stages of fermentation. Inevitably, however, it also eliminates some of the nutrients from the fermentation system, and therefore, a certain percentage of fresh medium needs to be added after the dialysis operation. This helps to “purify” the environment for the bacteria to grow and produce. Furthermore, the ability of *B subtilis* XGL to produce adenosine was restored after the dialysis operation. This is due to the fact that adenine leaves the fermentation system during membrane-coupled dialysis and, accordingly, inhibition of the purine synthesis pathway by adenine (Smith et al., 1994) is removed and adenosine production is restored. A precipitous drop in biomass volume occurred after each membrane-coupled dialysis operation. This was due to the loss of biomass during the dialysis process, and it is assumed that this may also be the reason for the stepwise decrease in biomass.

## Conclusion

The current methods of adenine production mainly include chemical synthesis and enzymatic catalysis. In this study, the enzymatic method was combined with the fermentation method and successfully achieved the conversion of adenine by adding AdHy crude enzyme solution during the production of fermented adenosine. The overall fermentation system was also permeabilized by coupling the fermentation system to a ceramic membrane. The final total adenine yield was 14 ± 0.25 g/L, the highest yield of adenine fermentation to date. The data shows that the dialysis of the fermentation system resulted in a significant increase in the viability of the bacterium and recovery of the productivity of the bacterium. This indicates that by using a coupled ceramic membrane to dialyze the whole fermentation system, the factors that are detrimental to the growth and production of the bacterium can be effectively removed from the fermentation system, and the growth capacity of the bacterium can be restored to a certain extent.

## Data Availability

The original contributions presented in the study are included in the article/Supplementary Material, further inquiries can be directed to the corresponding author.
