# Supplementary material for: Process study of ceramic membrane-coupled mixed-cell fermentation for the production of adenine
Source: Front Bioeng Biotechnol. 2022 Aug 10;10:969668. doi: 10.3389/fbioe.2022.969668 (PMC9399796; doi:10.3389/fbioe.2022.969668)
Supplement: Supplementary file 1 [file Table1.DOC]

**Table S1 *E.coil* BL21-ADH medium**

| component | Content（g/L） | sterilization temperature |
| --- | --- | --- |
| **Culture medium for strain activation** | | |
| Yeast extract | 0.5 | 0.01 Mpa，121℃，20min |
| Tryotone | 1 |
| Nacl | 1 |
| Agar powder | 25 |
| **Induced expression medium** | | |
| Glucose | 30 | 0.01 Mpa，115℃，15 min |
| Yeast extract | 8 |
| Tryotone | 4 |
| (NH_4_)_2_ SO_4_ | 3 |
| K_2_HPO_4_·3H_2_O | 4 |
| Citric acid | 1 |
| MgSO_4_·7H_2_O | 1 |
| MnSO_4_ | 10 mg/L |
| FeSO_4_·7H_2_O | 10 mg/L |
| VB_1_、VB_3_、VB_7_ | 1 mg/L |

**Table S2 *B. Subtilis* XGL medium**

| **Culture medium for strain activation** | | |
| --- | --- | --- |
| Yeast extract | 0.5 | 0.01 Mpa，121℃，20 min |
| Tryotone | 1 |
| Nacl | 1 |
| Agar powder | 25 |
| **seed culture medium** | | |
| Glucose | 30 | 0.01 Mpa，115℃，15 min |
| Yeast extract | 8 |
| Tryotone | 5 |
| (NH_4_)_2_ SO_4_ | 8 |
| Sodium glutamate | 3 |
| KH_2_PO_4_ | 3 |
| Histidine | 0.5 |
| MgSO_4_·7H_2_O | 0.5 |
| Xanthine | 200 mg/L |
| V_H_ | 2 mg/L |
| VB_1_、VB_3_、VB_7_ | 1 mg/L |
| **fermentation culture medium** | | |
| Glucose | 30 | 0.01 Mpa，115℃，15 min |
| Sodium glutamate | 12 |
| Yeast extract | 10 |
| KH_2_PO_4_· 3H_2_O | 6 |
| CaCl_2_ | 2 |
| Hypoxanthine | 20 |
| MgSO_4_·7H_2_O | 5 |
| (NH_4_)_2_ SO_4_ | 10 |
| Histidine | 0.5 |
| FeSO_4_·7H_2_O | 20 mg/L |
| MnSO_4_ | 10 mg/L |
| VB_1_、VB_3_、VB_7_ | 5 mg/L |
| Xanthine | 0.2 |
| **Continuous flow of nutrients** | | |
| Yeast extract | 10 | 0.01 Mpa，115℃，15 min |
| Histidine | 0.7 |
| Xanthine | 0.2 mg/L |
| VB_1_、VB_3_、VB_7_ | 1 mg/L |

**Table S3 Strain and plasmid information**

| **plasmid** | **Relevant characteristics** | **Sources** |
| --- | --- | --- |
| pET-28a | A shuttle expression vector of *E. coli*, Km^R^, T7 promoter | Laboratory stock |
| pET-28a-*Rihc* | pET-28a containing *Rihc* gene at the *EcoR* I and *Hind* IIIsites, Km^R^ | This work |
| **Strain** | **Relevant characteristics** | **Sources** |
| *Bacillus Subtilis* XGL | The adenosine producing strain (8-AGr, His^-^, Xan^-^, SG^r^) | Laboratory stock |
| *E. coli* DH5α | F^-^, φ80d*lacZ*ΔM15 Δ(lac*ZYA*-*argF*) U169*recA1* *endA1 hsdR17*(r_k_^-^, m_k_^+^) *deoR* *supE44*λ^-^ *thi-1* *gyrA*96 *relA1* | Laboratory stock |
| *E. coli* BL21（DE3） | F，ompT，hsdS B (r B - m B - )，gal(λcI857，ind1，Sam7，nin5，lacUV5-T7gene1)，dcm(DE3) | Laboratory stock |
| *E. coli* BL21-ADH | *E. coli* BL21 derivative harboring pET-28a-*Rihc* | This work |

**Table S4 List of primers involved in the construction of strains**

| **primer** | **sequence（5’-3’）** |
| --- | --- |
| pET-28a-S | TAATACGACTCACTATAGGG |
| pET-28a-A | TGCTAGTTATTGCTCAGCGG |

**Table S5 Orthogonal experiment of ultrasonic parameters**

| **experiment** | **power（W）** | **Duty cvele（%）** | **Time（min）** | **enzymatic activity（U/mL）** |
| --- | --- | --- | --- | --- |
| 1 | 130 | 38 | 7 | 172 |
| 2 | 130 | 42 | 8 | 182 |
| 3 | 130 | 45 | 9 | 185 |
| 4 | 150 | 38 | 7 | 179 |
| 5 | 150 | 42 | 8 | 192 |
| 6 | 150 | 45 | 9 | 193 |
| 7 | 170 | 38 | 7 | 190 |
| 8 | 170 | 42 | 8 | 207 |
| 9 | 170 | 45 | 9 | 203 |

**
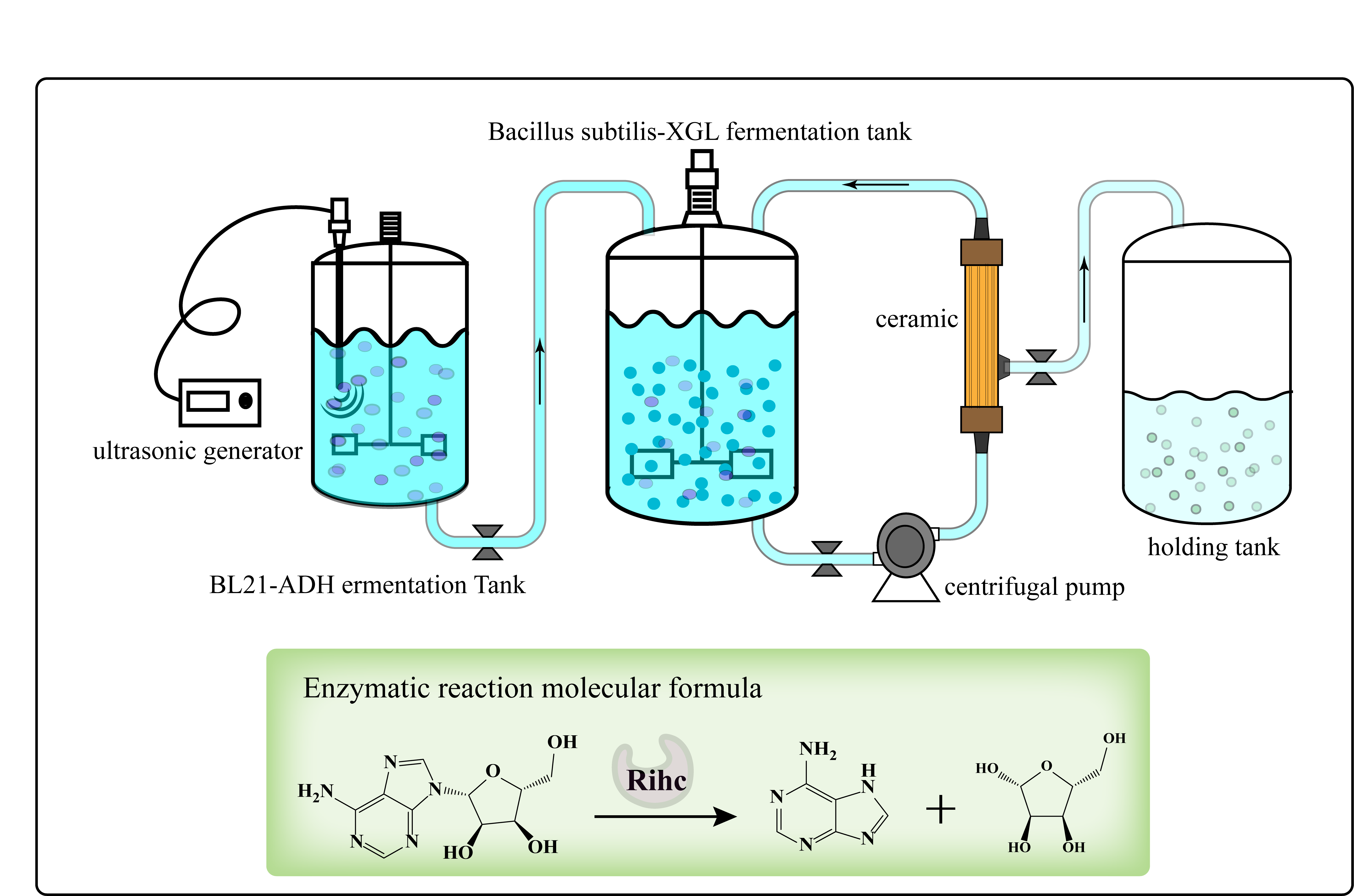
**

**Figure. 1** **Schematic diagram of the production of adenine by ceramic membrane coupled mixed cell fermentation**


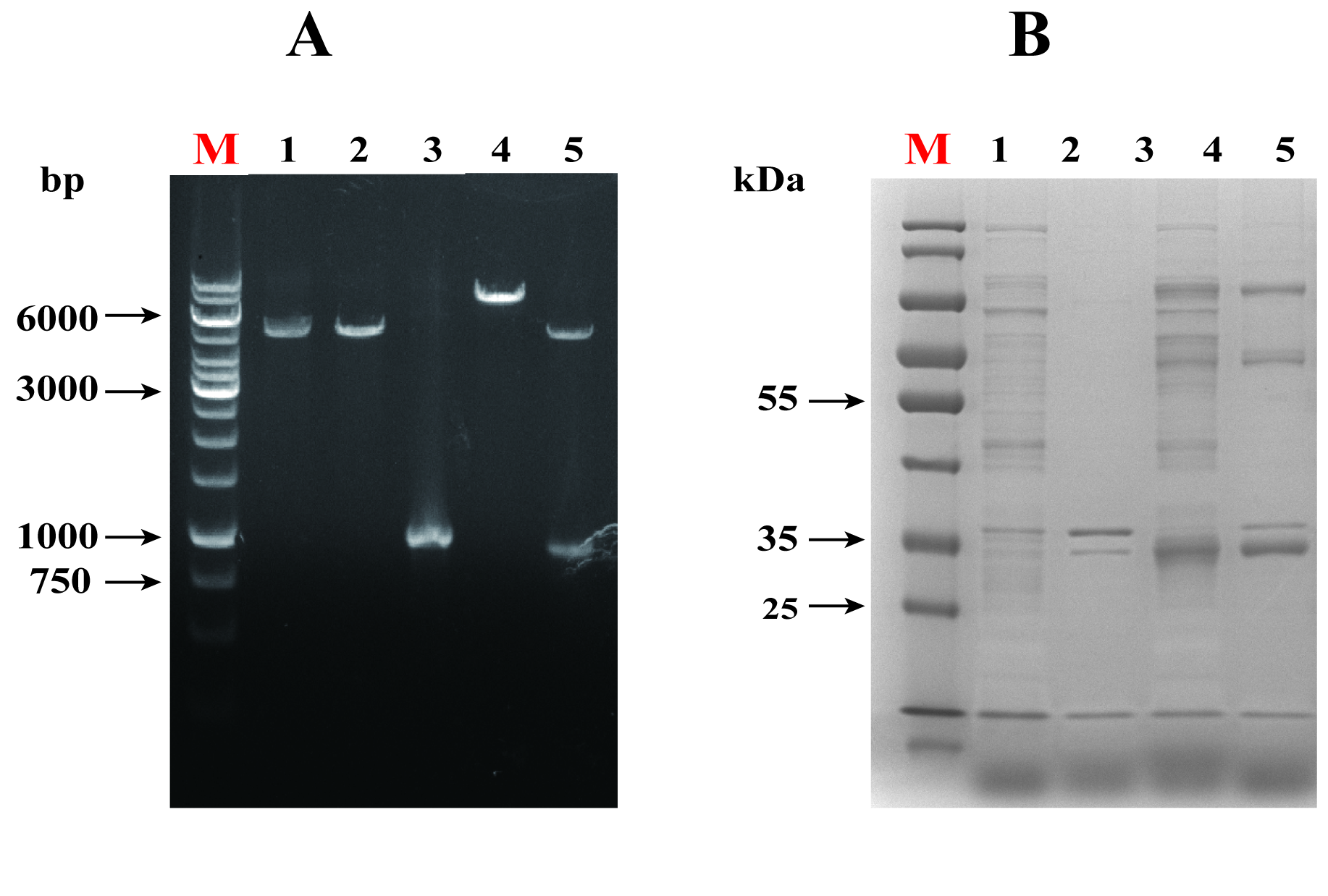
**Figure. 2** **alidation of Recombinant Expression of Nucleoside Hydrolase *Rihc***

(A)Restriction map of recombinant plasmid. M: DNA marker; 1:EcoR Ⅰ single enzyme digestion of pET-28a; 2:pET-28a was digested by EcoR Ⅰ and Hind Ⅲ. 3: target gene fragment; 4: EcoR I single enzyme digestion of pET-28a-Rihc; 5: pET-28a-Rihc was digested with EcoR I and Hind Ⅲ. (B) SDS-PAGE analysis of the recombinant protein. M: protein marker; 1: E. coli pET-28a cell lysate supernatant sample; 2: E. coli pET-28a cell lysate precipitation sample; 3: E. coli pET-28a- Rihc cell lysate supernatant sample; 4 : Sedimentation in cell lysate of E. coli pET-28a-Rihc.

**

**

**Figure. 3 Changes in enzyme activity and biomass during induction culture**

**
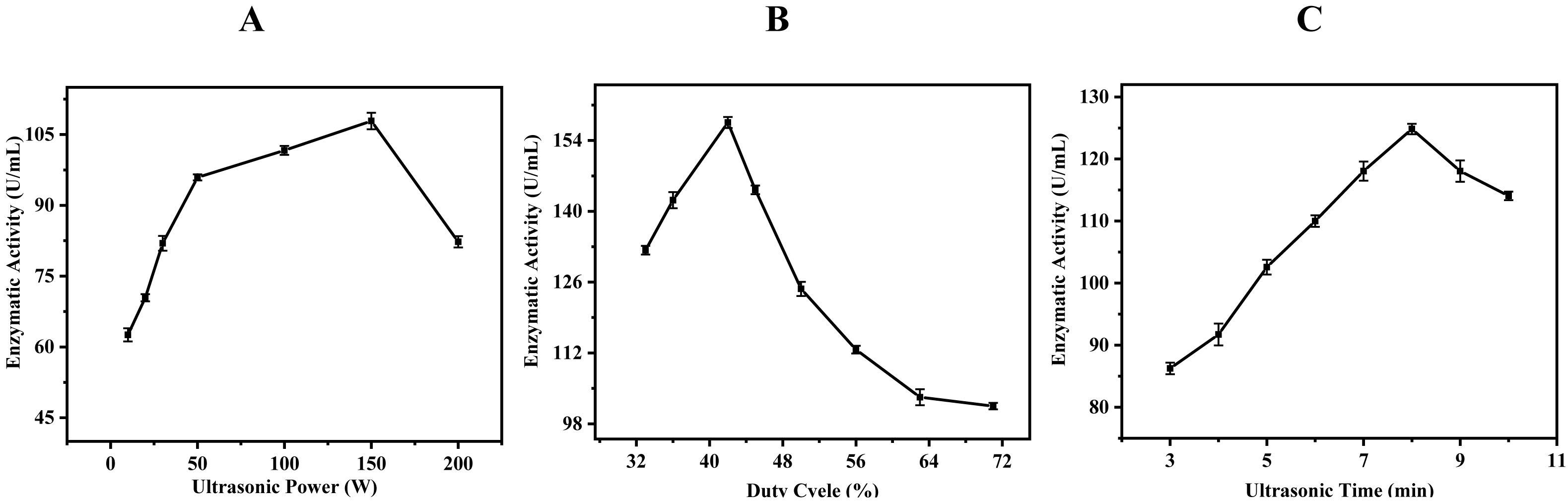
**

**Figure. 4 Single factor experiment of ultrasonic parameters**

A: Effect of ultrasonic power on enzyme activity; B: Effect of Duty Ratio on Enzyme Activity; C: Effect of ultrasonic time on enzyme activity

**
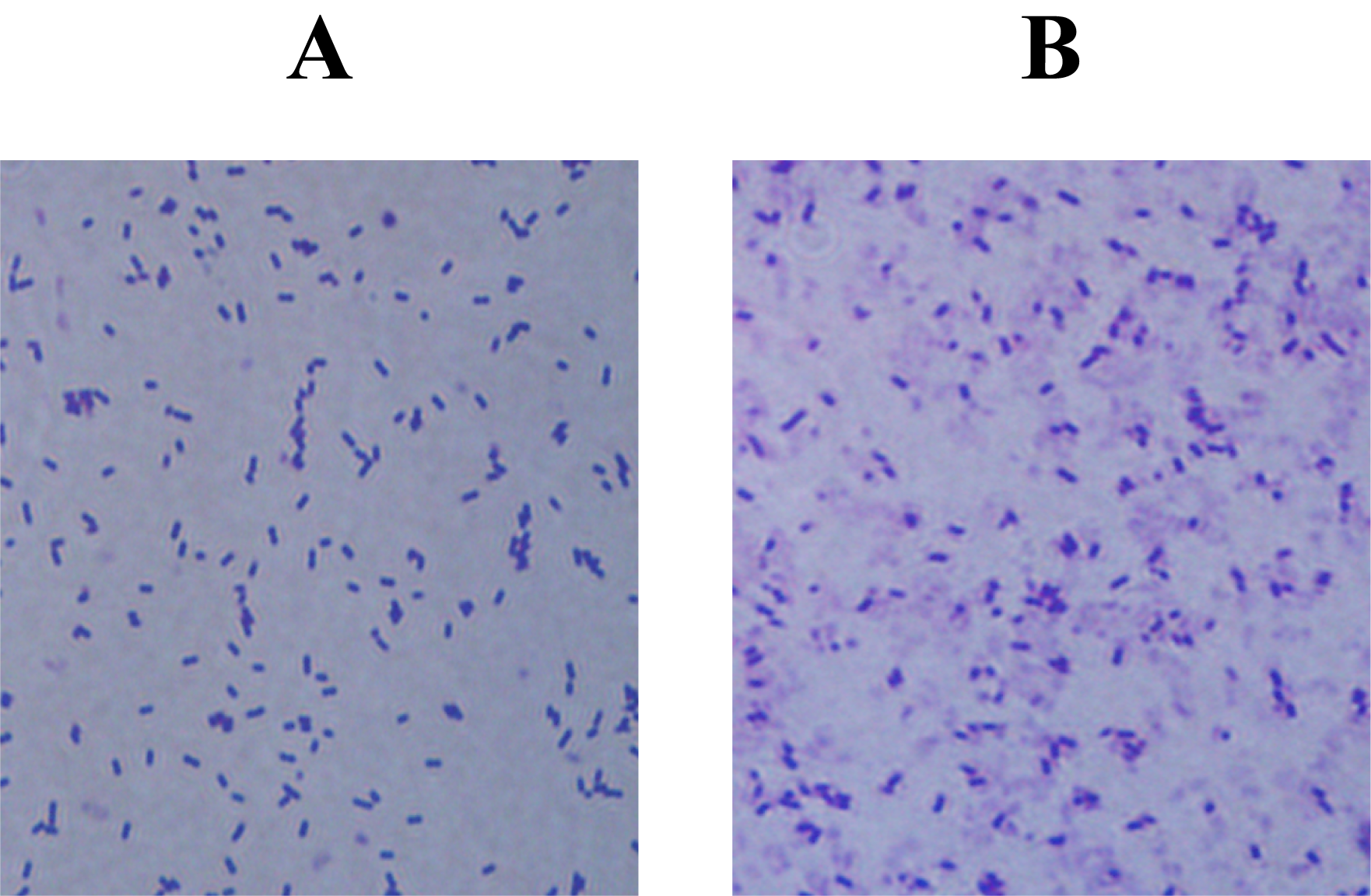
**

**Figure. 5 Morphological changes in *E. coli* BL21-ADH under optimized ultrasound conditions**

A: Normal bacterial morphology; B: Changes in bacterial morphology under ultrasound treatment**
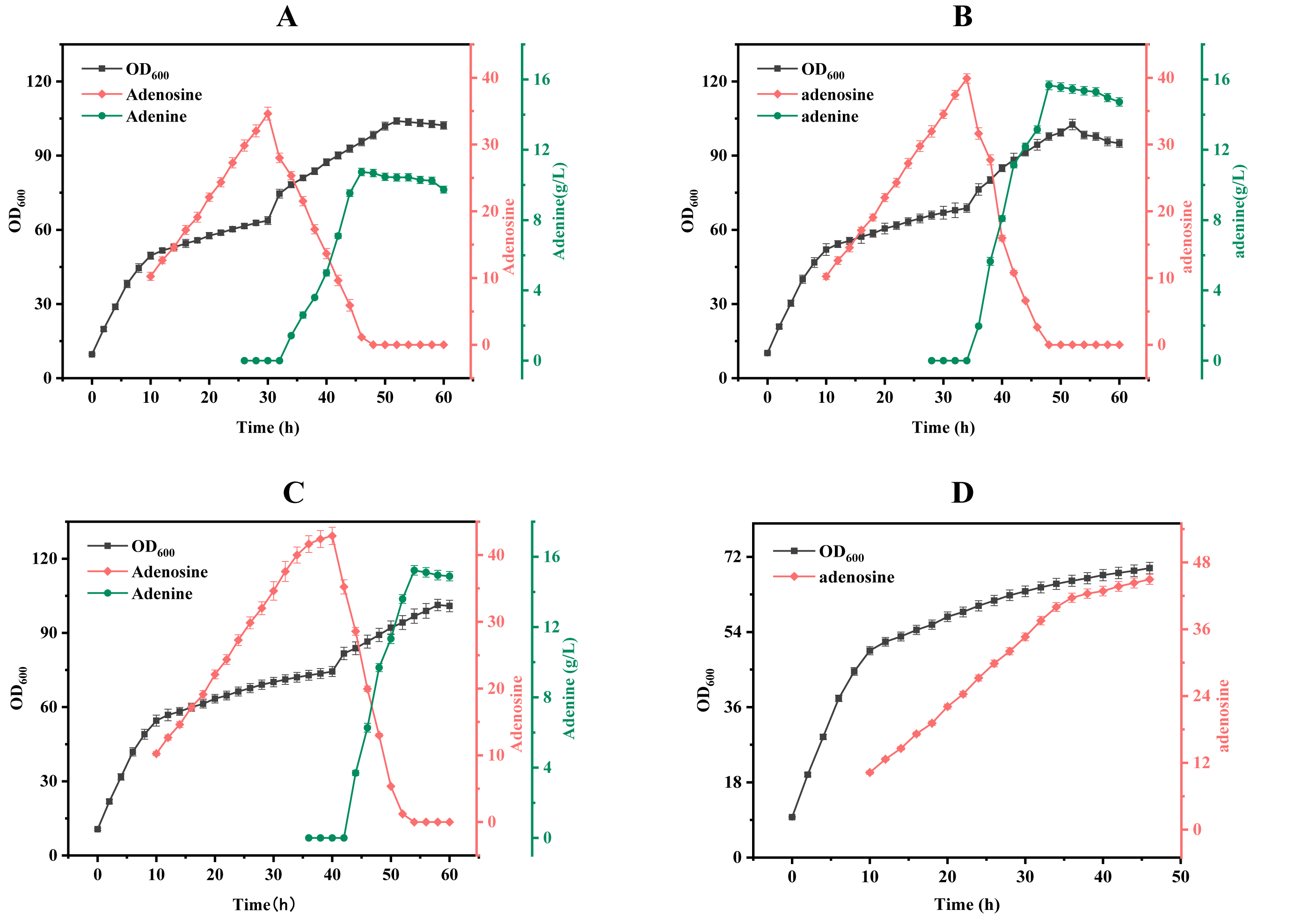
**

**Figure. 6 Effect of Different Mixing Time on Fermentation Results**

Mixed ratio: 13 % of Bacillus subtilis XGL fermentation system. (A): Changes of cell volume, adenosine and adenine production during 30 h mixing operation; (B): Changes of cell volume, adenosine and adenine production during 35 h mixing operation; (C): Changes of cell volume, adenosine and adenine production during 40 h mixing operatio; (D): Changes of cell volume and adenosine production during normal fermentation.

**
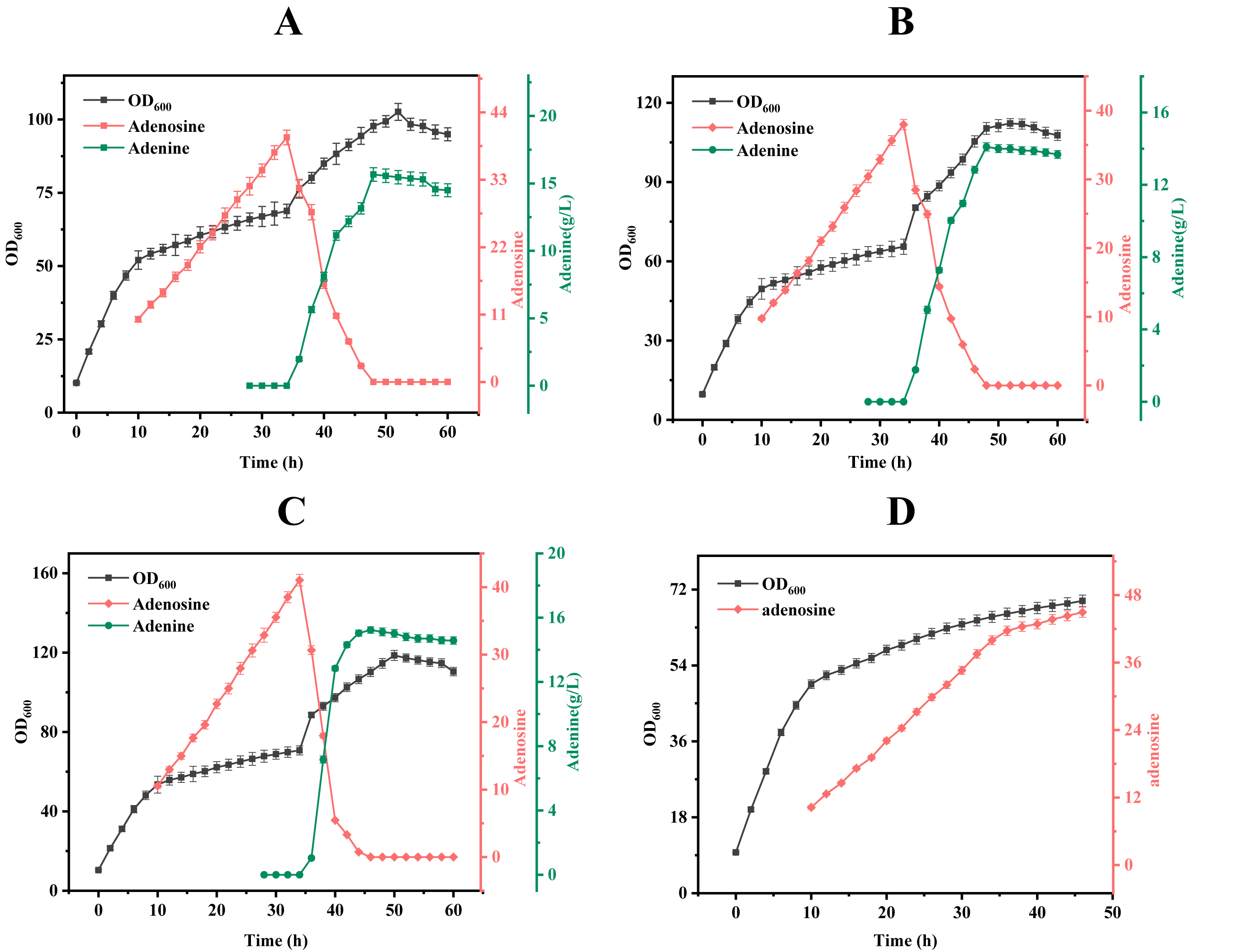
**

**Figure. 7 Effect of Different Mix Proportion on Fermentation Results**

Mixed time: B. subtilis XGL fermented to 35 h. (A): When the mixed ratio was 6 % of B. subtilis XGL fermentation system, the changes of cell volume, adenosine and adenine production were observed; (B): When the mixed ratio was 13 % of B. subtilis XGL fermentation system, the changes of cell volume, adenosine and adenine production were observed; (C): When the mixed ratio was 20 % of B. subtilis XGL fermentation system, the changes of cell volume, adenosine and adenine production were observed; (D): Changes of cell volume and adenosine production during normal fermentation.

**
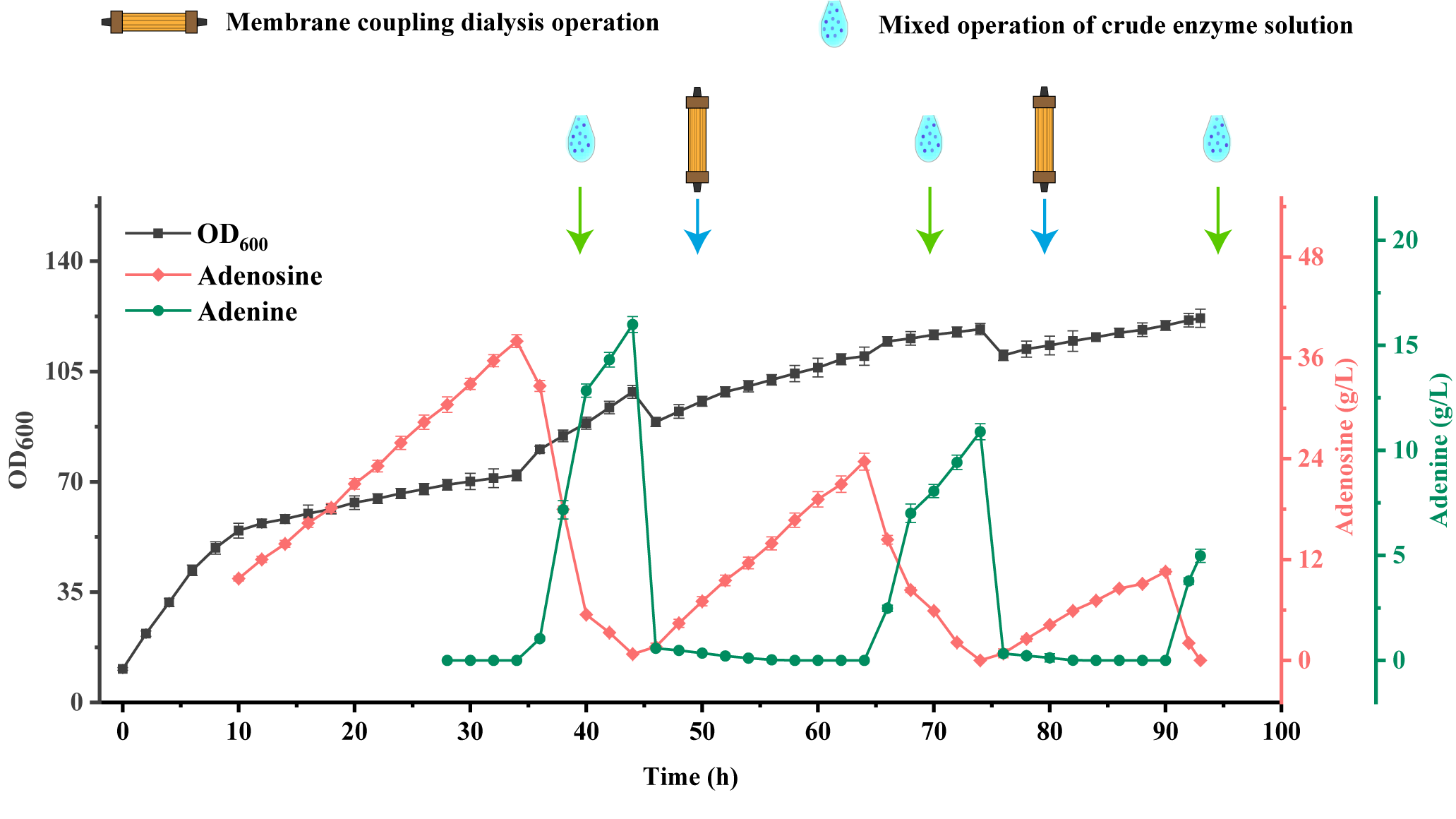
**

**Figure. 8 Changes of Cell Volume, Adenosine and Adenine Production inBacterial and Enzyme Mixed Coupling Ceramic Membrane Fermentation**
